# Correlation of cytokine level with the severity of severe fever with thrombocytopenia syndrome

**DOI:** 10.1186/s12985-016-0677-1

**Published:** 2017-01-13

**Authors:** Miao-Miao Liu, Xiao-Ying Lei, Hao Yu, Jian-zhi Zhang, Xue-jie Yu

**Affiliations:** 1 School of Public Health, Shandong University, Jinan, 250012 China; 2 School of Medicine, Fudan University, Shanghai, 200032 China; 3 School of Health Professions, University of Texas Medical Branch, Galveston, Texas 77555-0609 USA; 4 Department of Pathology, University of Texas Medical Branch, Galveston, Texas 77555-0609 USA

**Keywords:** Severe fever with thrombocytopenia syndrome (SFTS), Cytokine storm

## Abstract

**Background:**

Severe fever with thrombocytopenia syndrome (SFTS) was an emerging hemorrhagic fever that was caused by a tick-borne bunyavirus, SFTSV. Although SFTSV nonstructural protein can inhibit type I interferon (IFN-I) production Ex Vivo and IFN-I played key role in resistance SFTSV infection in animal model, the role of IFN-I in patients is not investigated.

**Methods:**

We have assayed the concentration of IFN-α, a subtype of IFN-I as well as other cytokines in the sera of SFTS patients and the healthy population with CBA (Cytometric bead array) assay.

**Results:**

The results showed that IFN-α, tumor necrosis factor (TNF-α), granulocyte colony-stimulating factor (G-CSF), interferon-γ (IFN-γ), macrophage inflammatory protein (MIP-1α), interleukin-6 (IL-6), IL-10, interferon-inducible protein (IP-10), monocyte chemoattractant protein (MCP-1) were significantly higher in SFTS patients than in healthy persons (*p* < 0.05); the concentrations of IFN-α, IFN-γ, G-CSF, MIP-1α, IL-6, and IP-10 were significant higher in severe SFTS patients than in mild SFTS patients (*p* < 0.05).

**Conclusion:**

The concentration of IFN-α as well as other cytokines (IFN-γ, G-CSF, MIP-1α, IL-6, and IP-10) is correlated with the severity of SFTS, suggesting that type I interferon may not be significant in resistance SFTSV infection in humans and it may play an import role in cytokine storm.

## Background

Severe fever with thrombocytopenia syndrome (SFTS) is an emerging hemorrhagic fever in East Asia that is caused by a tick-borne bunyavirus, SFTSV [1]. The major clinical manifestations of SFTS included acute fever (≥38 °C), thrombocytopenia, leucopenia, gastrointestinal symptoms, central nervous system (CNS) symptoms, and multiple organ dysfunctions [1–3]. The case fatality rate of the disease ranged from 12 to 30% in China and is even higher in Japan [1, 4]. However, the pathogenic mechanism of the disease was not well studied. Previous studies suggested that a cytokine storm might be associated with the development of the disease [5–7].

Cytokines are a group of small proteins which are essential for fighting off infections. However, overproduction of cytokines could trigger a dangerous situation known as a cytokine storm. The nonstructural protein of SFTSV has been described to inhibit type I interferon (IFN-I) production in cell culture [8, 9]. Mouse is naturally resistant to SFTSV infection, but IFN-α receptor (IFNAR) deficient mouse is highly susceptible to SFTSV infection [10]. These studies suggested that IFN-I may play an important role in resistance SFTSV infection. However, the role of IFN-I in resistance SFTSV infections has not yet been investigated in humans although the role of other cytokines has been well studied by several reports. The aim of this study is to determine the concentration of IFN-α, a subtype of IFN-I as well as the concentration of other cytokines in SFTS patients and in the healthy population. By analyzing the differences in cytokine concentrations between severe SFTS patents and mild SFTS patients and between SFTS patients and the healthy population, we attempted to determine the role of IFN-α and other cytokines in the pathogenesis of SFTS.

## Methods

### Patient sera and clinical information

The SFTS patients included in this study had met one or more of the following criteria: (1) SFTSV was isolated from the patient’s serum, (2) SFTSV RNA was detected from the patient’s serum by a quantitative reverse-transcriptase polymerase chain reaction (RT-PCR) and (3) seroconversion or a 4-fold increase of antibody titers was detected between the acute and convalescent sera of the same patient. The sera of SFTS patients used in this study were obtained during the acute phase of the disease (within 2 weeks since onset). The sera of healthy persons were obtained from the persons who came to the hospital for physical examination. In accordance with the illness severity, the patients were divided into severe and mild groups [2, 3, 6]. Severe SFTS cases were defined as patient who required admission to an intensive care unit and met at least one of the following criteria: (1) acute lung injury (ALI) /acute respiratory distress syndrome (ARDS), (2) heart failure, (3) acute renal failure, (4) encephalitis, (5) shock, (6) septicemia, (7) disseminated intravascular coagulation (DIC), or (8) death.

### Multiplex cytokine assay

The concentrations of serum cytokines were tested with BD™ human soluble protein master buffer kits and BD™ CBA flex sets according to the manufacturer’s instructions (BD Bioscience-PharMingen, San Diego, CA). The cytokines assayed in this study included tumor necrosis factor (TNF-α), granulocyte colony-stimulating factor (G-CSF), interferon-γ (IFN-γ), IFN-α, macrophage inflammatory protein (MIP-1α), interleukin-6 (IL-6), IL-10, interferon-inducible protein (IP-10), monocyte chemoattractant protein (MCP-1), and regulated upon activation normal T cell expressed and secreted factor (RANTES).

### Statistical analysis

The statistical analysis was performed using SPSS software 18.0 for Windows. Means for continuous variables were compared using independent-group Student’s *t* tests when the data were normally distributed; otherwise, the Wilcoxon rank sum test was used. The categorical variables were compared with *χ* ^2^ test. A *p*-value <0.05 was considered statistically significant.

## Results

### Patient information

Fifty SFTS patients who were confirmed to be infected by SFTSV previously were enrolled in this study [11]. The mean age was 61.2 (SD = 10.9) years old, and 17 (34.0%) of them were male. In addition to the patients, thirty-eight age and sex matched healthy individuals were also enrolled. The mean age of healthy individuals was 62.7 (SD = 13.2) years old, and 14 (36.8%) of them were male. There were no significant differences between patients and normal subjects on the distribution of age and gender (Table 1). Among the fifty patients, thirty-six of them were mild patients and fourteen were severe patients. There was no difference on gender distribution between the severe patients (male, 28.6%) and the mild patients (male, 36.1%). However, the severe patients were significantly older than the mild patients (67.0 VS 59.5, *p* = 0.003) (Table 2).

**Table 1:** Comparison of age, gender and cytokine concentrations between SFTS patients and healthy population^a^

Characteristic	Patients (*n* = 50)	Healthy Individuals (*n* = 38)	*p* Value
Sex, male, n (%)	17 (34.0)	14 (36.8)	0.782
Age, year, mean (SD)	61.2 (10.9)	62.7 (13.2)	0.554
TNF-α	1.2 (0.6–1.6)	0.0 (0.0–0.3)	0.001
G-CSF	7.6 (5.5–18.7)	4.0 (3.5–4.4)	0.027
IFN-γ	10.8 (4.6–34.5)	2.2 (1.7–2.6)	0.001
IFN-α	14.1 (6.5–29.5)	2.4 (1.9–2.8)	0.001
MIP-1α	16.4 (5.0–46.7)	0.1 (0.0–2.0)	0.045
IL-6	28.1 (10.7–70.5)	4.0 (3.3–6.6)	0.001
MCP-1	156.7 (88.9–407.9)	37.7 (20.7–61.3)	0.005
IL-10	1228.4 (88.8–3960.9)	1.0 (0.8–1.7)	0.002
IP-10	2718.7 (1389.9–5278.9)	65.1 (37.6–168.5)	0.001
RANTES	4676.3 (2982.4–7555.5)	7220.0 (5468.7–100115.6)	0.003

Unit: pg/ml; ^a^Values are listed as median range unless otherwise noted; SD, standard deviation

**Table 2:** Comparison of age, gender and cytokine concentrations between severe and mild SFTS patients^a^

Characteristic	Mild patients (*n* = 36)	Severe patients (*n* = 14)	*p* Value
Sex, male, n (%)	13 (36.1)	4 (28.6)	0.613
Age, year	59.5 (49.3–65.0)	67 (62.0–73.5)	0.003
TNF-α	1.2 (0.7–1.6)	1.3 (0.5–4.0)	0.314
G-CSF	6.9 (5.3–10.6)	20.1 (11.7–49.1)	0.001
IFN-γ	8.8 (3.9–25.1)	54.3 (6.2–90.4)	0.005
IFN-α	11.0 (5.3–18.8)	26.9 (14.3–138.3)	0.001
MIP-1α	12.1 (2.5–30.7)	56.4 (20.6–222.0)	0.001
IL-6	18.3 (7.9–45.8)	121.2 (47.5–272.4)	0.001
MCP-1	141.7 (85.4–339.9)	227.8 (118.6–1185.4)	0.115
IL-10	1188.7 (87.7–2436.2)	3917.0 (106.9–11025.9)	0.210
IP-10	2178.0 (1260.8–3715.6)	5385.2 (3362.3–8394.3)	0.003
RANTES	4855.4 (3070.1–7739.9)	4195.5 (2728.6–6199.0)	0.612

Unit: pg/ml; ^a^Values are listed as median range unless otherwise noted

### Cytokine concentrations

#### Comparison of cytokine concentrations between SFTS patients and healthy population

Except for RANTES, the concentrations of all tested cytokines including TNF-α, G-CSF, IFN-γ, IFN-α, MIP-1α, IL-6, IL-10, IP-10, and MCP-1 were significantly higher in SFTS patients than those in healthy individuals (*p* < 0.05) (Table 1). The concentration of IFN-γ, IFN-α, TNF-α, G-CSF, MCP-1, and IL-6 were moderately increased in SFTS patients than in the healthy population with 1 to 7 fold increases, whereas, the concentration of MIP-1α, IP-10, and IL-10 were extremely increased in SFTS patients than that in healthy individuals with 42 to 1228 fold increases. RANTES was the only cytokine that was decreased in SFTS patients than in healthy persons (4676.3 VS7220.0, *p* = 0.003).

#### Comparison of cytokine concentrations between severe and mild SFTS patients

The results showed that severe SFTS patients were significantly older than the mild patients. In order to confirm whether age is an influencing factor for the concentrations of cytokines, the healthy individuals were divided into two parts according to their ages. There was no significant difference between the two parts on the concentration of cytokines (fifty percent of them were older than 65 years, data not shown). The concentrations of G-CSF, IFN-α, IFN-γ, MIP-1α, IL-6, and IP-10 were significantly increased in severe SFTS patients than in mild patients (*p* < 0.05); whereas the concentrations of TNF-α, MCP-1, IL-10 and RANTES did not significantly differ between the two groups of patients (Table 2). Severe SFTS patients exhibited a moderate 1.6 to 6.7-fold increase in cytokine concentrations when compared to mild SFTS patients.

## Discussion

Cytokines, a class of small molecule proteins with a broad range of biological activities, are mainly synthesized and secreted by immune cells [12]. According to their functions, cytokines can be divided into the following categories: chemokine, interferon, interleukin, colony stimulating factor, tumor necrosis factor, transforming growth factor and growth factor. They act through receptors and play an important role in host immune response to infections and cancer. Viral infections can stimulate the body to produce immune responses, including the innate immune response and adaptive immune response. TNF-α and IFNs are involved in the innate immune response, which is the first line of defense against viral infections. In the subsequent adaptive immune response, IL, CSF, chemokine and other cytokines play an important role [13].

The concentrations of most cytokines have been reported previously [5–7]. Our results were consistent with previous studies, indicating that the cytokine level of G-GSF, IFN-γ, IL-6, IL-10, TNF-α, MIP-1α, MCP-1, and IP-10 were significantly higher in SFTS patients than in the healthy controls. Moreover, the levels of G-CSF, IFN-γ, MIP-1α, IL-6, and IP-10 were significantly higher in severe SFTS patients than the mild patients.

Interferons are glycoprotein secreted by mononuclear leukocytes and lymphocytes in response to pathogens, such as viruses and bacteria, or tumor cells [14]. Based on the type of receptor, human interferons have been classified into three major types: interferon type I, interferon type II and interferon type III. In a typical scenario, a virus-infected cell will release interferons causing nearby cells to heighten their anti-virus defenses. IFN-α proteins produced by leukocytes [15] is a member of the type I interferon family, a multi-gene cytokine family that includes IFN-α, IFN-β, IFN-ε, IFN-τ, IFN-κ, IFN-ω, IFN-δ and IFN-ζ in humans [16]. All IFN-I bind to a cell surface receptor complex, IFN-α receptor (IFNAR), consisting of IFNAR1 and IFNAR2 chains [17, 18]. IFN-I has been found in all mammals and similar molecules have been found in fish, reptile and birds [17, 18]. IFN-I has diverse effects on innate and adaptive immune cells during infections, directly and/or indirectly through the induction of other mediators [19]. Normal mice are resistant to SFTSV infection, but IFNAR deficient mice became highly susceptible to SFTSV infection, suggesting the importance of IFN-I in resistance to SFTSV infection [10]. However, IFN-I has been shown to cause immunopathology in some acute viral infections, such as influenza virus infection [19]. The changes of IFN-I content during different viral infections varied. The level of IFN-α was significantly elevated in the serum of SARS and Hantavirus pulmonary syndrome patients [20, 21]. However, another study indicated that the IFN-I response was not notable during hantavirus infection [22]. Given the importance of IFN-I against viral infections or in immunopathology of viral infections, it is surprising that only one article reported the IFN-α2 concentration in the serum of SFTS patients [2]. Our original hypothesis was that IFN-I should be low in severe SFTS patients than in mild SFTS patients because it had the function in defending against viral infection. However, our results showed the opposite, suggesting that high concentrations of IFN-α in SFTS patient could not control SFTSV, which may actually involve in cytokine storm. It is interesting to note that in vitro studies indicated that the non-structural proteins of SFTSV inhibits the generation of type I interferon to promote viral replication [8, 9, 23]. However, our results indicated that the level of IFN-α is significantly higher in SFTS patients than healthy persons. Moreover, IFN- α is also significant higher in severe SFTS patients than in mild SFTS patients. A previous study also indicated that severe SFTS patients had higher SFTSV viral loads in serum than mild SFTS patients [24]. Combining the results of our study and the previous study, we concluded that higher SFTSV viral load stimulate stronger IFN-α production in vivo, suggesting that non-structural proteins of SFTSV may be not important for inhibition of the production of type I INFs in vivo. Our result was different on IFN-α compared to a previous study, which showed no significant difference between severe and mild patients (2). The concentration discrepancy may be caused by different collection stage or storage condition.

Our results showed that IFN-γ was also correlated to the severity of SFTSV infection, which is consistent with a previous study [2]. However, another study indicated that levels of IFN-γ in non-severe cases were lower than values detected in healthy individuals [6].

G-CSF stimulates bone marrow to generate granulocytes and release them into the blood stream. It can stimulate the survival, proliferation, differentiation, and function of neutrophil precursors and mature neutrophils, which have phagocytosis and chemotaxis function against infection [25]. However, when the body was infected with SFTSV, the white blood cell count would progressively decrease [26–28]. G-CSF may be produced to increase white blood cells against SFTSV infection. Our study indicated that G-CSF is significantly increased in SFTS patients and it is correlated to the severity of SFTSV infection.

Interleukins are mainly expressed by leukocytes and their main functions are to promote the development and differentiation of T and B lymphocytes, and hematopoietic cells [29]. IL-6 plays an important role in inducing B cells to differentiate into plasma cells to produce antibody [30]. IL-10 down-regulates the immune response after virus infection by inhibiting IFN-γ production, antigen presentation, and macrophage production of IL-1, IL-6, and TNF-α. IL-10 induces activated B cells to secrete large amounts of IgG, IgA, and IgM. Thus, IL-10 may play an important role in the amplification of humoral responses [31]. IL-6 and IL-10 are usually increased in viral infections. IL-6 and IL-10 were significantly elevated in Chikungunya fever patients compared to uninfected persons [32]. IL-10 level was significantly elevated at the early stage of Ebola infection [33]. The role of IL-10 in inhibiting antigen-stimulated T cell proliferation supports the assumption that a T cell–mediated response is critical for survival of Ebola virus infected patients [33, 34]. Both IL-6 and IL-10 are significantly increased in SFTS patients and lL-6, but not IL-10 is correlated to the severity of SFTSV infection.

TNF-α is a monocyte-derived cytotoxin that can directly kill tumor cells and has no obvious toxicity to normal cells. By binding to the corresponding receptor, it participates in host antiviral response [35]. Consistent with previous studies, we demonstrate that the concentration of TNF-α is extremely low in healthy individuals (median = 0, range: 0-0.3 pg/ml); however, even in severe patients, its concentration is only 1.3 pg/ml (range: 0.5-4.0). There was no significant difference between the severe SFTS patients and the mild SFTS patients which suggested that TNF-α may not play an important role in the progress of infection as previously expected.

MIP-1α, MCP-1, and IP-10 are chemokines, a family of small signaling proteins mainly secreted by white blood cells. The major role of chemokine is to act as a chemoattractant to guide the migration of cells [36]. During the processes of immune surveillance, chemokine can regulate cells of the immune system, such as directing lymphocytes to the lymph nodes so they can screen for invasion of pathogens by interacting with antigen-presenting cells residing in these tissues [37]. Our study showed that the increase of both MIP-1 and IP-10 are significantly correlated to the severity of SFTSV infection. Several previous studies have also reported increase of these cytokines in SFTS patients [2, 6].

RANTES, a known chemoattractant for monocyte and T cells, is produced by many cell types, including endothelial cells and macrophages [38]. RANTES have an essential role in activation and proliferation of antigen-specific T cells [39]. It was the only indicator we studied that had a higher level in healthy subjects. Furthermore, its concentration was decreased in severe patients compared to the mild patients. This result was completely opposite with other cytokines. In a previous study, the level of RANTES decreased in SFTS patient which was consistent with our result [5]. However, in another study the concentration of RANTES in patients’ sera was obviously increased compared to healthy persons [6]. The concentration discrepancy of RANTES in different studies may be caused by collecting the samples in different stage of infection.

Cytokine storm is a potentially fatal immune reaction consisting of a positive feedback loop between cytokine and white blood cells, with highly elevated levels of various cytokines [40]. Studies found that a variety of pathogen infections can cause a cytokine storm, which may cause acute respiratory distress syndrome and multiple organ failure [5, 41–43]. Previous studies demonstrated that SFTSV infection induced a cytokine storm with abnormally expressed cytokine profiles, which might be associated with the disease severity [5, 6, 44]. Consistent with our study, the elevated cytokines in those above studies included IP-10, G-CSF, IL-6, IL-10, MCP-1, and MIP-1α. However, the differences of TNF-α and IFN-γ concentration between severe and mild SFTS patients were varied in different studies. Deng, et al showed that the concentrations of TNF-α and IFN-γ were significantly higher in severe cases than non-severe cases [6]. However, there were people who also indicated that the production of TNF-α and IFN-γ during the acute phase were only slightly elevated in severe cases [5]. In this study, most of the cytokines including G-CSF, IFN-γ, IFN-α, MIP-1α, IL-6, and IP-10 were correlated to the severity of SFTSV infection.

## Conclusion

Our results indicated that IFN-α as well as other cytokines including G-CSF, IFN-γ,, MIP-1α, IL-6, and IP-10 were correlated to the severity of SFTSV infection, suggesting that type I interferon may not play a significant role in resistance SFTSV infection in humans and it may play an import role in cytokine storm involved in the pathogenesis of SFTS.

## Abbreviations

G-CSF: granulocyte colony-stimulating factor
IP-10: interferon-inducible protein
MCP-1: monocyte chemoattractant protein
MIP-1α: macrophage inflammatory protein
RANTES: Regulated upon activation normal T cell expressed and secreted factor
RT-PCR: Quantitative reverse-transcriptase polymerase chain reaction
SFTS: Severe fever with thrombocytopenia syndrome
